# Three-dimensional computed tomography morphometric analysis of lumbar endplates in Chinese young adults

**DOI:** 10.3389/fsurg.2026.1703513

**Published:** 2026-04-07

**Authors:** Yukai Cui, Wei Wang, Xiaohao Sun, Haoran Zhang, Wen Yin, Xilong Cui, Wei Jiao

**Affiliations:** 1 Department of Orthopaedic, Fuyang Hospital Affiliated With Anhui Medical University (Fuyang People's Hospital), Fuyang, China; 2 Department of Orthopaedic, Fuyang Hospital Affiliated With Bengbu Medical University (Fuyang People's Hospital), Fuyang, China; 3 Department of Orthopaedic, Anhui Provincial Clinical Medical Research Center for Spinal Deformities, Fuyang, China; 4 School of Mechatronics Engineering and Automation, Shanghai University, Shanghai, China

**Keywords:** coronal endplate, interbody fusion device, lumbar fusion, sagittal endplate, three-dimensional CT

## Abstract

**Purpose:**

To analyze the morphological characteristics of the lumbar 4 (L4)- Sacral 1 (S1) endplates in the Chinese young adults lumbar spine based on three-dimensional (3D) computed tomography (CT) scans, providing a reference for the design and application of intervertebral fusion devices.

**Methods:**

We collected data from 33 patients with healthy lumbar spines and performed lumbar CT scans and 3D model reconstructions of the vertebral endplates. Sagittal and coronal dimensions of the concave areas of the endplates were measured from the L4 superior endplate to the S1 superior endplate, as was the depth of the most concave point in the concave areas of the endplates. The endplate morphology was classified and compared between sexes.

**Results:**

In the sagittal plane, uniformly concave endplate, 20.6%; asymmetric concave, 32.7%; flat-bottomed concave, 12.1%; and flat endplate, 34.5%; in the coronal plane, uniformly concave endplate, 32.1%; asymmetric concave, 0.6%; flat-bottomed concave, 52.7%; and flat endplate, 14.5%. There were no statistically significant differences in the sagittal and coronal morphology of the lower lumbar endplates between sexes (*p* > 0.05). The depth of the most concave point in the lower endplate depression area of the L4 and L5 vertebral bodies was greater than that of the upper endplates, while the depth of the most concave point in the lower endplate depression area of the L4, L5, and S1 intervertebral spaces was smaller than that of the upper endplates of the superior vertebral bodies, with statistically significant differences (*p* < 0.05). There were no statistically significant differences in the maximum depth of depression in the sagittal and coronal planes of the endplates between different sexes at each segment (*p* > 0.05).

**Conclusions:**

The sagittal plane morphology of the lower lumbar endplate is mostly concave, whereas the sagittal plane morphology of the S1 endplate is mostly flat. Asymmetric endplates are less common in the coronal plane, and the endplates in the coronal plane are mostly symmetrically distributed. Sex has no significant effect on the morphology of the endplates.

## 1 Introduction

Lumbar degenerative diseases, including spondylolisthesis, disc herniation, and spinal stenosis, are increasingly prevalent in younger populations worldwide, posing a growing clinical challenge despite advances in conservative management (1). Lumbar interbody fusion surgery, a common and effective surgical method for treating these conditions, has been widely applied in clinical practice to restore spinal stability, maintain intervertebral height, and promote fusion (2). The intervertebral cage, as the core component of the procedure, plays a crucial role and is used to fix the relative positions of the vertebral bodies, reconstruct the stability of the spine, increase the rate of bone graft fusion, and promote the fusion of the intervertebral disc and its surrounding structures. However, there are post-operative complications such as settlement and displacement of the interbody fusion device, which cause a series of clinical symptoms and lead to secondary surgery (3). These postoperative complications are typically caused by poor compatibility between the interbody fusion device and the complex shape of the vertebral endplate. The vertebral endplate, as the primary interface for axial load transfer and maintaining biomechanical stability in the spine, plays a crucial role in compatibility (4, 5).

Selecting the appropriate intervertebral fusion device plays an important role in maintaining the anatomical height of the intervertebral space and reconstructing the physiological curvature of the lumbar spine. Furthermore, precise matching of the surface curvature and the end plate anatomical morphology can effectively optimize the stress distribution and avoid stress concentration to reduce post-operative complications such as settlement, displacement, and pseudarthrosis formation (6, 7). Additionally, the diverse morphology of the vertebral endplate and the relatively simple surface structure of the fusion device may result in a mismatch between the intervertebral disc and vertebral body, affecting contact, pressure distribution, and load transfer. This could lead to device displacement, improper subsidence, and reduced interbody fusion effectiveness (8). Therefore, it is important to understand the morphological characteristics of the vertebral endplate and to ensure detailed measurement of the parameters of the endplate to optimize the design of the intervertebral endcage, to maximize the joint area of the intervertebral endcage and the endplate.

However, there are few reports on the morphology of the lumbar endplate, and most only have data measurements from two-dimensional lateral lumbar x-rays, lumbar magnetic resonance imaging (MRI), or computed tomography (CT) (9, 10). The classification of endplate morphology is also limited. This study, therefore, aimed to investigate endplate morphology through CT scans, three-dimensional (3D) models, anatomical measurements, and statistical analysis of the adult lumbar endplate. The outcome of the study was determination of the influence of different sexes on endplate morphology to provide guidance for lumbar intervertebral body fusion.

## 2 Methods

This study was approved by the Ethics Committee of our hospital [Permit No. (2022)33]. This study retrospectively analyzed the imaging data of patients who underwent reconstruction of 3D lumbar CT images in our outpatient clinic from April 2022 to April 2024. Basic information of patients was recorded, including name, sex, age, height, weight, etc.

### 2.1 Inclusion and exclusion criteria

Patients were included if they were aged 18–45 years, displayed normal lumbar alignment on imaging, and had no history of spinal disease. Exclusion criteria were expanded to include: (1) spinal fractures, tumors, or prior surgery; (2) degenerative conditions like spondylolisthesis or significant disc herniation; (3) congenital anomalies such as scoliosis (Cobb angle > 10°) or lumbosacral transitional vertebrae; and (4) poor CT image quality that impeded 3D reconstruction.

### 2.2 CT and 3D scans

**Figure 1:**
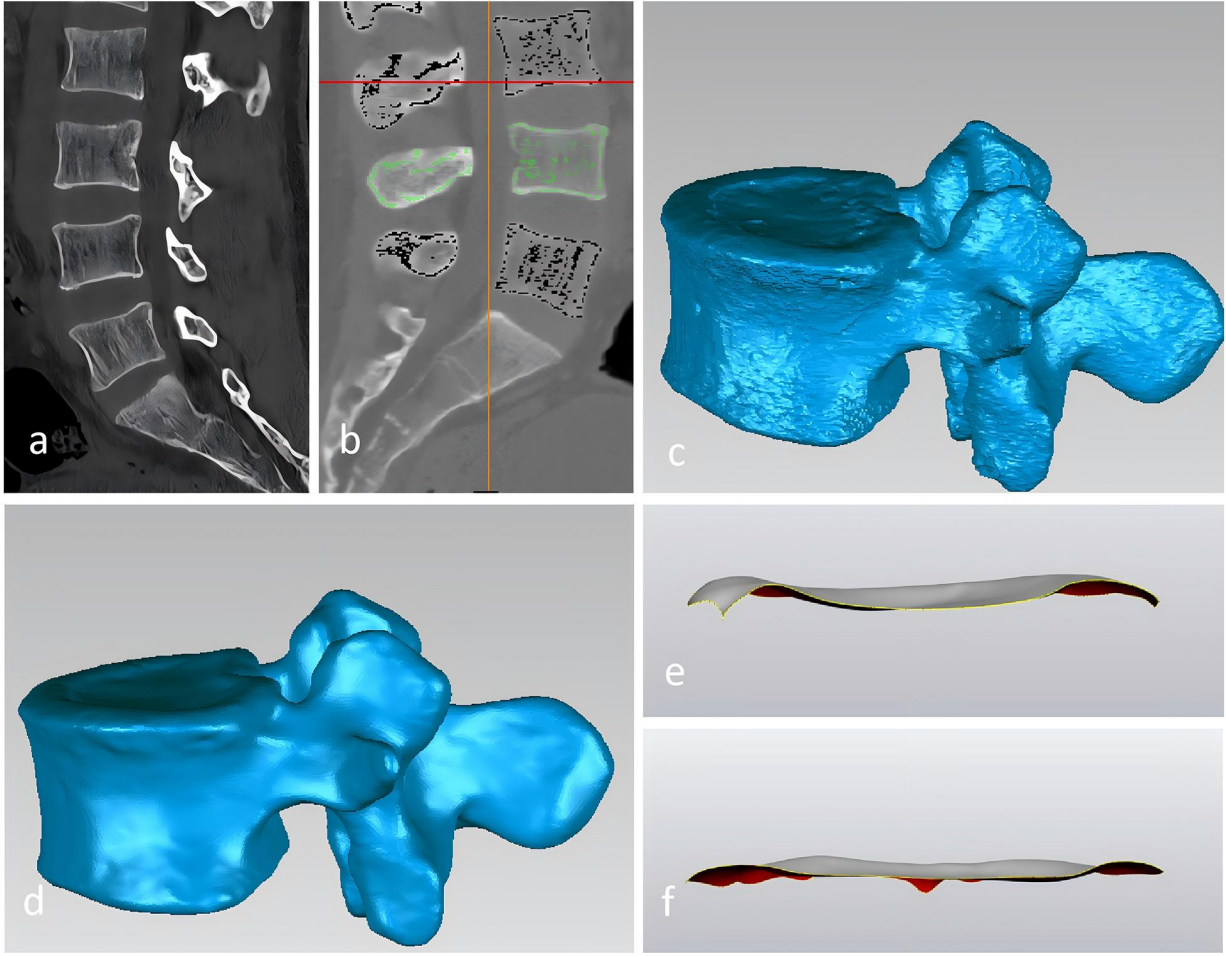
Image processing workflow for one representative patient. **(a)** Raw 2D DICOM slice from PACS. **(b)** After segmentation in Mimics. **(c)** Before smoothing in Geomagic Wrap. **(d)** After smoothing. **(e)** Sagittal endplate map. **(f)** Coronal endplate map.

Lumbar spine CT scans were performed using a Siemens SOMATOM 64-slice spiral CT scanner (Siemens Healthineers, Erlangen, Germany). The scanning parameters were optimized for bony structures: tube voltage of 120 kV, automatic tube current modulation (CARE Dose4D), and a pitch of 0.9. The images were reconstructed with a slice thickness of 0.75 mm and a matrix of 512 *512. The resulting high-resolution DICOM data were then imported into Mimics (Materialize, 21.0) for 3D segmentation and reconstruction. To ensure high-precision boundary detection, manual segmentation under expert supervision was performed using specific bone thresholds, followed by region-growing algorithms to maintain vertebral integrity. The resulting vertebrae were saved in STL format and imported to Geomagic Wrap (3D Systems, 2021) to smooth the images and remove noise. Finally, the models were introduced into 3-Matic (Materialize, 15.0), where curvature analysis was utilized to manually separate the vertebral endplate from the vertebral body. The upper and lower endplates of the L4 and L5 vertebrae and the upper endplates of S1 were anatomical measured by the length measurement tool on the software (accuracy of 0.01 mm) (Figure 1).

### 2.3 Anatomical data

**Figure 2:**
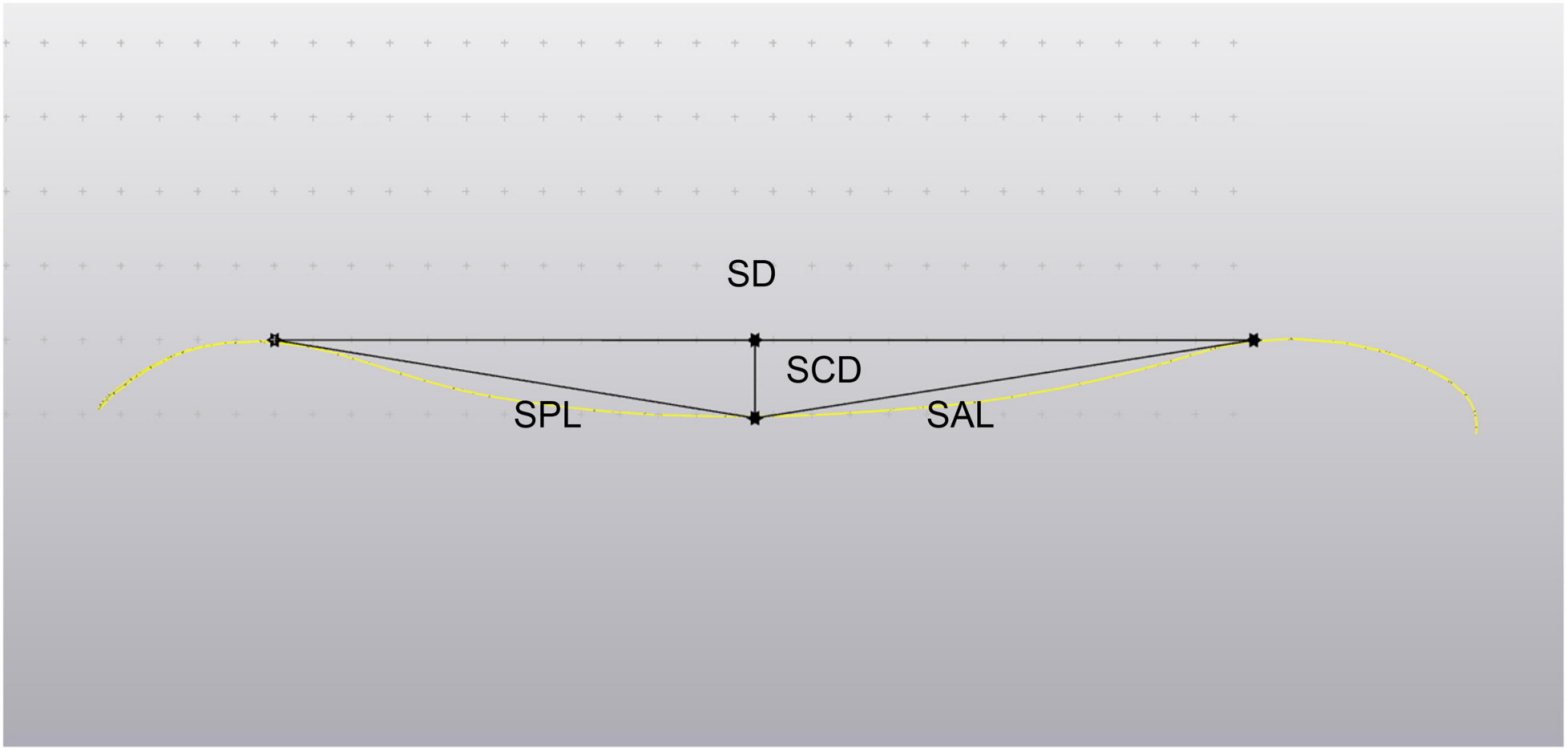
Schematic representation of the sagittal morphometric parameters of the vertebral endplate. The Sagittal Diameter (SD) was defined by connecting the highest points of the anterior and posterior margins. The Sagittal Concavity Depth (SCD) was recorded as the maximum vertical distance from the SD line to the deepest point of the endplate surface. Additionally, the Sagittal Posterior Length (SPL) and Sagittal Anterior Length (SAL) were measured by connecting the point of maximum depression to the posterior and anterior margins, respectively.

**Figure 3:**
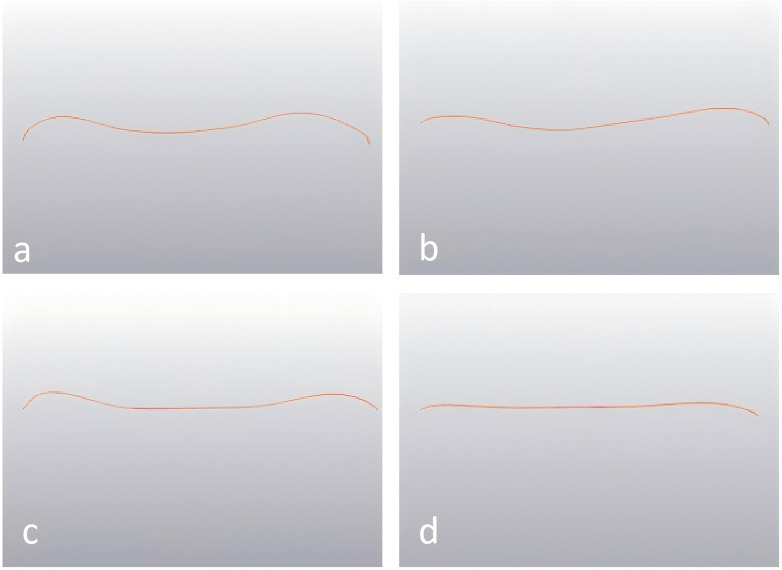
Endplate positions. **(a)** Uniform recessed endplate: SCD (CCD) ≥ 1 mm and 1.3 > SPL/SAL or SAL/SPL (CRL/CLL or CLL/CRL) > 1; **(b)** asymmetric recessed endplate: SCD (CCD) ≥ 1 mm and long SPL/SAL or SAL/SPL (CRL/CLL or CLL/CRL) ≥ 1.3; **(c)** flat bottom recessed endplate: SCD (CCD) ≥ 1 mm and flat surface of both sides; **(d)** flat: SCD (CCD) < 1 mm.

First, the superior and inferior endplates of the L4–L5 vertebra and the superior endplate of the S1 vertebra were stripped to obtain an independent 3D model of the endplate. Second, the sagittal plane and coronal plane of the endplate were segmented to obtain the curved surface of the sagittal plane and coronal plane. For morphometry, the Sagittal Diameter (SD) was defined by connecting the highest points of the anterior and posterior margins. The Sagittal Concavity Depth (SCD) was recorded as the maximum vertical distance from the SD line to the deepest point of the endplate surface. Additionally, the Sagittal Posterior Length (SPL) and Sagittal Anterior Length (SAL) were measured by connecting the point of maximum depression to the posterior and anterior margins, respectively. In the coronal plane, the Coronal Diameter (CD) was defined as the baseline connecting the superior-most points of the left and right lateral margins. To determine the Coronal Concavity Depth (CCD), a perpendicular line was dropped from the CD baseline to the point of maximum depression on the endplate surface. Furthermore, the Coronal Right Length (CRL) and Coronal Left Length (CLL) were measured by connecting this deepest point of concavity to the highest points of the right and left lateral rims, respectively (Figure 2). According to the length of the SCD, CCD, the position of the largest depression of the end plate, and the shape of the curved surface, the sagittal and coronal forms were divided into uniform concave, asymmetric concave, flat concave, and flat surfaces as follows: SCD (CCD) < 1 mm was flat; SCD (CCD) ≥ 1 mm and 1.3 > SPL/SAL or SAL/SPL (CRL/CLL or CLL/CRL) > 1 was uniform concave; SCD (CCD) ≥ 1 mm and long SPL/SAL or SAL/SPL (CRL/CLL or CLL/CRL) ≥ 1.3 was asymmetric concave; and SCD (CCD) ≥ 1 mm and flat surface of both sides was flat bottom depression type (Figure 3). The data were measured and recorded by two senior orthopedic surgeons with more than 5 years of experience. Any differences were clarified by the corresponding author.

### 2.4 Statistical analysis

Statistical analyses were performed using SPSS 27.0 (IBM, Armonk, NY). Quantitative variables were expressed as mean ± standard deviation (SD) and 95% confidence intervals (CIs). The normality of data distribution was assessed using the Shapiro–Wilk test. Continuous variables following a normal distribution were compared between sexes using independent-sample t-tests. Categorical data, such as the distribution of endplate morphology types, were analyzed using the Chi-square test or Fisher's exact test as appropriate. Cramer's V was utilized as the effect size measure for categorical comparisons. A *p*-value < 0.05 was considered statistically significant.

Additionally, the reliability of the 3D segmentation and manual measurements was assessed using the Intraclass Correlation Coefficient (ICC). Two senior orthopedic surgeons independently performed measurements on 10 randomly selected patients (50 endplates) to evaluate inter-observer reliability. One observer repeated the measurements after a two-week interval to evaluate intra-observer reliability. A two-way random-effects model with absolute agreement was employed, with ICC values > 0.75 considered to reflect excellent reliability.

## 3 Results and discussion

### 3.1 Demographic characteristics and clinical data

Based on the inclusion and exclusion criteria, 33 patients were enrolled, including 19 males and 14 females. All patients underwent 3D reconstruction imaging of the lumbar L4–L5 segment CT, and the demographic characteristics and clinical data of the included patients are shown in Table 1. Statistical analysis confirmed that there were no significant differences in age or BMI between the male and female subgroups (*p* > 0.05).

**Table 1:** Demographics and clinical characteristics of the patients.

Characteristic	*n* = 33
Age (years ± SD)	35.3 ± 6.7
Sex (*n*, %)	
Male	19 (57.6%)
Female	14 (42.4%)
BMI (kg/m^2^ ± SD)	24.52 ± 2.69

BMI: body mass index (kg/m^2^).

### 3.2 Measurement reliability and reproducibility

Reliability analysis showed that the ICC values of major morphological parameters, including SCD, CCD, SD, and CD, were all above 0.75. This confirmed the high stability and reproducibility of the manual segmentation and measurement procedures.

### 3.3 Endplate morphology distribution

In this study, 165 endplates were explored in 33 patients. A sagittal endplate shape was recorded in 20.6% of patients (34/165); asymmetric depressed endplate, 32.7% (54/165); flat bottom depressed endplate, 12.1% (20/165); and flat endplate, 34.5% (57/165) (Table 2). The recorded coronal endplate pattern was uniform depressed in 32.1% (53/165) of patients; asymmetric depressed endplate, 0.6% (1/165); flat bottom depressed endplate, 52.7% (87/165); and flat endplate, 14.5% (24/165) (Table 3).

**Table 2:** Form distribution of the L4–S1 sagittal endplate of the lumbar spine.

Segment	Uniformly concave *n* (%)	Asymmetric concave *n* (%)	Flat-bottomed concave *n* (%)	Flat type *n* (%)
L4 sup	9 (27.3)	7 (21.2)	7 (21.2)	10 (30.3)
L4 inf	15 (45.5)	9 (27.3)	7 (21.2)	2 (6.1)
L5 sup	2 (6.1)	17 (51.5)	3 (9.1)	11 (33.3)
L5 inf	8 (24.2)	18 (54.5)	2 (6.1)	5 (15.2)
S1 sup	0	3 (9.1)	1 (3.0)	29 (87.9)

**Table 3:** Form distribution of the L4–S1 coronal plane of the lumbar spine.

Segment	Uniformly concave *n* (%)	Asymmetric concave *n* (%)	Flat-bottomed concave *n* (%)	Flat type *n* (%)
L4 sup	0	1 (3.0)	19 (57.6)	13 (39.4)
L4 inf	21 (63.6)	0	12 (36.4)	0
L5 sup	2 (6.1)	0	20 (60.6)	11 (33.3)
L5 inf	10 (30.3)	0	23 (69.7)	0
S1 sup	20 (60.6)	0	13 (39.4)	0

### 3.4 Maximum depression depth

**Figure 4:**
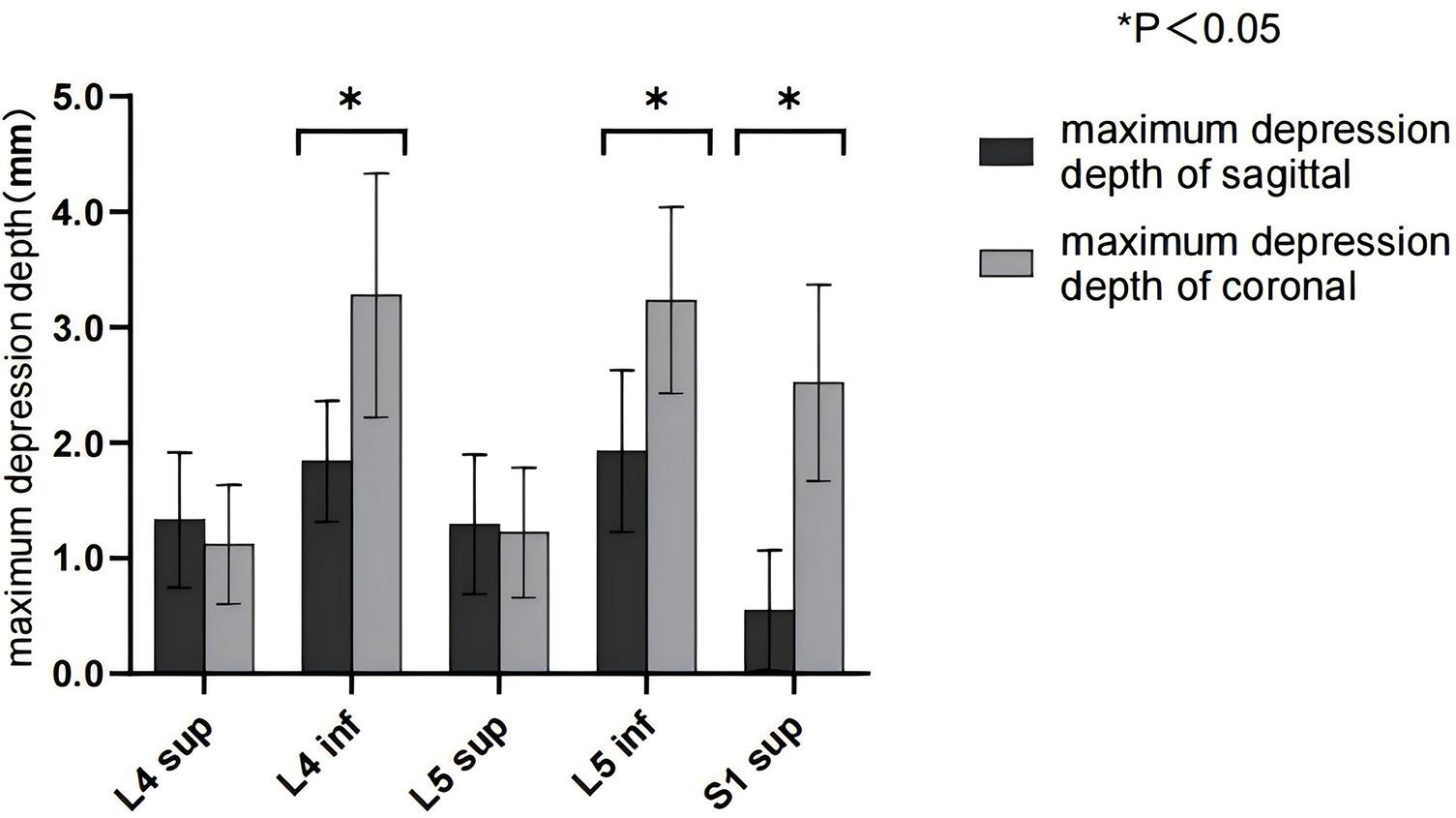
Maximum depression depth of the sagittal L4–S1 and coronal surfaces.

The maximum depression depth of the sagittal end plate and the coronal end plate of the lower lumbar spine was greater than that of the upper end plate and the upper end plate of L4 and S1 (*p* < 0.05, Figure 4).

### 3.5 Sex differences

There was no statistically significant difference in the sagittal and coronal endplate morphology between sexes (*p* > 0.05, Tables 4, 5). There was no statistical difference in the maximum depression depth of the same sagittal and coronal planes between the different sexes (*p* > 0.05, Tables 6, 7).

**Table 4:** Sagittal endplate morphology in different sexes.

Segment	Uniformly concave type	Asymmetric concave type	Flat-bottomed concave type	Flat type	X^2^	*p*(Fisher's exact)	Cramer's V
L4 sup							
male (*n* = 19)	4	5	2	8	_	0.14	0.41
female (*n* = 14)	5	2	5	2			
L4 inf							
male (*n* = 19)	7	6	4	2	_	0.61	0.28
female (*n* = 14)	8	3	3	0			
L5 sup							
male (*n* = 19)	1	11	1	6	_	0.83	0.19
female (*n* = 14)	1	6	2	5			
L5 inf							
male (*n* = 19)	4	11	1	3	_	0.94	0.10
female (*n* = 14)	4	7	1	2			
S1 sup							
male (*n* = 19)	0	2	1	16	_	1.00	0.17
female (*n* = 14)	0	1	0	13			

**Table 5:** Coronal endplate morphology in different sexes.

Segment	Uniformly concave type	Asymmetric concave type	Flat-bottomed concave type	Flat type	X^2^	*p*(Fisher's exact)	Cramer's V
L4 sup							
male (*n* = 19)	0	0	11	8	_	0.69	0.21
female (*n* = 14)	0	1	8	5			
L4 inf							
male (*n* = 19)	11	0	8	0	_	0.49	0.14
female (*n* = 14)	10	0	4	0			
L5 sup							
male (*n* = 19)	0	0	11	8	_	0.23	0.34
female (*n* = 14)	2	0	9	3			
L5 inf							
male (*n* = 19)	5	0	14	0	_	0.71	0.10
female (*n* = 14)	5	0	9	0			
S1 sup							
male (*n* = 19)	13	0	6	0	_	0.28	0.19
female (*n* = 14)	7	0	7	0			

**Table 6:** Maximum depression in the sagittal plane of the endplate by sex.

Segment	Male	Female	95% CI of difference	t	*p*	Cohen's d
L4 sup	1.32 ± 0.70	1.35 ± 0.41	−0.46,0.40	−0.15	0.89	−0.05
L4 inf	1.77 ± 0.56	2.06 ± 0.43	−0.66,0.08	−1.61	0.11	−0.57
L5 sup	1.33 ± 0.71	1.25 ± 0.44	−0.37,0.52	0.35	0.73	0.12
L5 inf	1.89 ± 0.73	1.98 ± 0.69	−0.60,0.42	−0.35	0.73	−0.12
S1 sup	0.63 ± 0.65	0.44 ± 0.25	−0.18,0.56	1.03	0.30	0.36

**Table 7:** Maximum depression in coronal sections of endplates by sex.

Segment	Male	Female	95% CI of difference	t	*p*	Cohen's d
L4 sup	1.07 ± 0.56	1.18 ± 0.46	−0.47,0.28	−0.53	0.60	−0.19
L4 inf	3.16 ± 0.99	3.43 ± 1.15	−1.03,0.49	−0.72	0.48	−0.25
L5 sup	1.12 ± 0.56	1.35 ± 0.55	−0.56,0.22	−1.15	0.26	−0.32
L5 inf	3.20 ± 0.82	3.28 ± 0.82	−0.66,0.51	−0.26	0.80	−0.09
S1 sup	2.73 ± 0.65	2.23 ± 0.54	−0.09,1.10	1.74	0.09	0.61

### 3.6 Discussion

The sagittal plane morphology of the lower lumbar endplate is mostly concave, whereas the sagittal plane morphology of the S1 endplate is mostly flat. Asymmetric endplates are rare in the coronal plane, where endplates are mostly symmetrically distributed. Sex has no significant effect on the morphology of the endplates.

The vertebral endplate is essential for overall stability of the spine. By evenly transferring the pressure of the upper vertebral body to the intervertebral disc, the surface shape of the bony endplate can disperse the localized pressure, especially the mild depression of the bony end disc on the surface of the endplate. This can increase the contact area with the intervertebral disc, prevent excessive pressure in a single site, and avoid premature degeneration or damage of the intervertebral disc.

When the fusion apparatus and the endplate are poorly matched, the contact area is significantly reduced (9). Matsumura et al. showed that the incidence of sedimentation of the interbody cage decreased significantly when the cage covers more than 30% of the endplate area (11). Buttermann et al. (12) tested three different biomechanical instruments (polymeric cages, modular interbody spacers, and tapered interbody cages) in 18 cadavers, showing that tight association between the interbody device and endplate significantly influences internal fixation stability and osteointegration. At present, the vertebral endplate is routinely trimmed to increase the contact area with the interbody fusion device to ensure a closer fit.

However, by studying the upper and lower end plates of the L3–L5 vertebrae in body specimens, Oxland et al. (13) showed that the average damage load in the local area decreased by 33% after removal of the hard endplate layer. These changes lead to uneven vertebral stress distribution and localized stress concentration, increasing the risk of vertebral injury and disc degeneration. Cheng et al. (14) performed biomechanical testing on eight vertebral endplates through the indentation test protocol and showed the average rigid reduction by 44% with 1-mm reduction in thickness and by 52% with 2-mm reduction. Strengthening the protection of the endplate during surgery and reducing the damage to the endplate can effectively reduce the risk of adjacent level disc degeneration and reduce the occurrence of vertebral pain (15, 16). These studies highlight the importance of intraoperative endplate retention. Therefore, in interbody fusion surgery, the selection of an appropriate fusion device should be based on the morphological characteristics of the endplate. The goal is to reduce intraoperative endplate damage and maximize the contact area between the device and the endplate. This approach helps maintain vertebral mechanical stability and normal spinal function, thereby reducing the incidence of post-operative complications.

The sagittal morphology of 165 endplates as well as the coronal morphology was studied and divided into four types: uniform depression, asymmetric depression, flat bottom depression, and flat depression. In the sagittal plane, the asymmetric concave and flat endplates accounted for 32.7% (54/165), and the ones accounted for 34.5% (57/165). The asymmetric concave endplate is mainly concentrated in the L4 segment lower endplate, L5 segment upper endplate, and L5 segment lower endplate. The S1 segment accounted for 50.9% of flat endplates. In a study of the sagittal plane morphology of the adult lower lumbar spine by MR, the S1 segment flat endplate accounted for most endplates, which is consistent with the results of this study. He X et al. (17) pointed out that the degree of lumbar disc degeneration is negatively correlated with the degree of endplate depression. The L5/S1 segment is a primary site of lumbar disc herniation, but the S1 superior endplate rarely exhibits a flat three-dimensional morphology. The authors suggest that the sagittal endplate morphology may be one of the influencing factors for lumbar intervertebral disc herniation. But this hypothesis still requires further investigation to be validated.

The lumbar endplate is not a regular elliptical or concave structure, and the median sagittal and coronal planes are different in shape (18). Studies on endplate morphology have focused on the sagittal plane of the endplate and its concavity; however, the morphological distribution of the coronal plane also has key effects on the biomechanical behavior of the spine and the adaptation of the interbody cage (19). Here, in the crown shape of the end plate, the asymmetric depressed end plate appeared less often; it was mainly uniformly depressed, flat bottom depressed, and flat ended, and the flat endplate only appeared in L4 and L5 endplates. Further quantitative analysis showed that the morphology of uniform depression, flat bottom depression, and flat endplates is roughly symmetrical, which indicates that the endplate form is symmetrical in the coronal plane. Compared with the diversity of the sagittal plane investigations, this feature may be the result of evolution to bilateral mechanical load. Van der Houwen et al. (20) measured endplate morphology by CT scanning technique, and confirmed that it was symmetrically distributed in the coronal plane.

The precise shape and geometric characteristics of the vertebral endplate are crucial for studying the biomechanics and morphology of the spine, and it is an important data reference for the design of interbody fusion devices. In this study, the maximum depression depth of the sagittal and coronal surfaces of the same vertebral body is less than the maximum depression depth of the lower terminal plate, while the maximum depression depth of the lower vertebral plate of the same vertebral space is less than the upper endplate of the lower vertebral body. Panjiabi and Van der Houwen found similar results (20, 21). Furthermore, Chen et al. (22) measured the sagittal depression depth of 97 patients and found that the maximum depression depth of the upper cervical endplate was also less than that of the lower endplate. Clinically, these morphological insights have direct implications for mitigating postoperative complications in lumbar interbody fusion, such as subsidence and pseudarthrosis. For instance, flat-shallow intervertebral spaces are associated with higher subsidence risks and lower fusion rates due to suboptimal stress transfer, as evidenced in biomechanical models (19). To address this, asymmetric cage designs can optimize load distribution and prevent such failures by better conforming to these depth variations. In addition, personalized interbody fusion cages manufactured using patient-specific CT reconstruction and 3D printing technology can achieve a larger endplate contact area, a higher bone fusion success rate, and a lower rate of surgical revision (23).

Sex has no obvious effect on the shape of the endplate and the maximum depth of the depression. This lack of difference may be attributed to similar biomechanical loads in both sexes, such as comparable axial compressive forces from daily activities and posture, which shape endplate morphology uniformly (9, 24). Genetic factors likely play a role, with no sex-specific variations in genes regulating bone remodeling, leading to analogous endplate contours despite overall vertebral size differences (25) While Singh et al. (26) found a correlation between sex and the endplate form and the maximum depression depth, this contrasts with Chen et al. (27), who found no differences in sagittal concave angle (SCA) and coronal concave angle (CCA) between sexes. Therefore, the endplate shape and the maximum depression depth of the endplate are not determined by sex. In addition, the maximum depression depths of the lower endplate of L4, L5 lower endplate, and S1 were statistically significant (*p* < 0.05). Therefore, in the design of interbody fusion devices, the shape of the upper and lower endplates, as well as differences between the maximum depression depth of the upper and lower end plates, should be considered. Additionally, symmetrical designs of the interbody fusion device should be avoided. A personalized design should be carried out according to the endplate anatomy data of each patient, so as to meet the needs of individualized treatment.

## 4 Limitations

There are some limitations to our study. First, the sample size of this study is small, so further large sample size studies are required to verify the morphology of the lower lumbar endplate. Second, although measurements followed standardized procedures, manual operations may introduce inter-observer variability. Third, the analysis based on 3D CT data did not use automatic segmentation, which could affect the accuracy and reproducibility of endplate boundary detection. Additionally, the 3D model lacks verification through cadaveric measurements, raising concerns about its absolute geometric accuracy. Finally, patients were selected as the normal lower lumbar spine population in this study; the morphology of lumbar endplate in patients with lumbar degenerative diseases was not compared, and their differences were not verified.

## 5 Conclusion

We established 3D reconstruction models of patients with computer-aided software to measure the anatomical structure of the sagittal plane and coronal plane of endplates. We proposed four types of endplates and refined the classifications thereof. Our results not only provide reference data for the design of intervertebral fusion devices, but also formulate preoperative end plate morphology for clinical surgical patients, to aid in the construction of personalized intervertebral fusion devices for patients and reduce the occurrence of post-operative complications.

## Data Availability

The raw data supporting the conclusions of this article will be made available by the authors, without undue reservation.
